# Cost-Effective Markers for Identifying Poor Glycemic Control in Type 2 Diabetes Mellitus: The Role of the Triglyceride-Glucose Index

**DOI:** 10.7759/cureus.88597

**Published:** 2025-07-23

**Authors:** Suheir Ereqat, Maha Sharabati, Mayar Abuhilal, Abedelmajeed F Nasereddin

**Affiliations:** 1 Biochemistry and Molecular Biology, Al-Quds University, Jerusalem, PSE; 2 Faculty of Medicine, Al-Quds University, Jerusalem, PSE; 3 Internal Medicine, St. Joseph Hospital, Jerusalem, PSE; 4 Al-Quds Bard College, Al-Quds University, Jerusalem, PSE

**Keywords:** glycemic control, insulin resistant, t2dm, tyg-bmi, tyg index

## Abstract

Background

Insulin resistance is a major risk factor for type 2 diabetes mellitus (T2DM), contributing to poor glycemic control and diabetic complications. This study aimed to investigate the correlation of the triglyceride-glucose (TyG) index, TyG-BMI index, and TG/HDL ratio with glycemic control.

Methods

A cross-sectional study was conducted from October 2023 to February 2024 at the Jericho Health Center, Palestine. A total of 240 T2DM patients were categorized into two groups based on glycemic control: good (HbA1c <7) and poor (HbA1c ≥7). Spearman correlation was used to assess the associations, and receiver operating characteristic (ROC) curve analysis was used to evaluate the predictive performance of the indices.

Results

Of 240 patients, 39% had good and 61% had poor glycemic control. The TyG and TyG-BMI indices were significantly higher (P<0.001) in those with poor glycemic control (5.0 vs. 4.8; 155 vs. 142, respectively). The TyG index cutoff was 4.8 (81% sensitivity, 55% specificity; AUC=0.744, p<0.001) while the TyG-BMI index cutoff was 162 (43.2% sensitivity, 76.3% specificity; AUC=0.591, p=0.018). The TyG index correlated positively with fasting plasma glucose (r=0.687), HbA1c (r=0.464), triglycerides (r=0.740), and cholesterol (r=0.254), and negatively with HDL-C (r=-0.200; all p<0.01). TyG-BMI correlated strongly with BMI (r=0.939) and weakly but significantly with systolic (r=0.204) and diastolic blood pressure (r=0.146). No significant correlation was found between TG/HDL and glycemic control.

Conclusion

Compared to TyG-BMI, the TyG index showed better diagnostic accuracy and stronger associations with metabolic markers, suggesting it may serve as a cost-effective and practical marker for evaluating glycemic control in T2DM patients. The low area under the curve (AUC) and sensitivity of TyG-BMI might limit its usefulness in this context. Establishing a population-specific cutoff based on inexpensive and routine laboratory measures enhances its practical utility in clinical settings.

## Introduction

Type 2 diabetes mellitus (T2DM) is a metabolic disease characterized by insulin resistance and chronic hyperglycemia, affecting approximately 90% of diabetes cases worldwide [1]. The global prevalence of type 2 diabetes mellitus (T2DM) is projected to increase from 9.3% in 2019 to 10.9% by 2045 due to sedentary lifestyles, aging, and obesity [2]. The top 10 countries with the highest prevalence of diabetes in the world include India, China, the USA, Indonesia, Japan, Pakistan, Russia, Brazil, Italy, and Bangladesh [3]. Diabetes contributes to a significant burden of chronic complications, which can be categorized into macrovascular (e.g., coronary artery disease, cerebrovascular disease, and peripheral artery disease) and microvascular (e.g., retinopathy, nephropathy, and neuropathy) complications [4]. In Palestine, an estimated 315,300 individuals aged 20 to 79 years were living with diabetes in 2024, including approximately 7,300 with type 1 diabetes. This number is expected to increase to 790,500 by 2050. There are about 95,200 individuals with undiagnosed diabetes in the same age group, and 13.6% of deaths among individuals aged 20-79 are attributed to diabetes-related causes [3]. Early detection and effective glycemic control are essential to mitigate diabetic complications. The World Health Organization (WHO) has recommended the use of glycosylated hemoglobin (HbA1c) as a screening test for people at high risk of diabetes, assessing long-term blood glucose levels and reflecting glycemic control over three months [5]. Maintaining HbA1c < 7% for both type 1 and type 2 diabetes is a target for the prevention of microvascular and macrovascular complications [6]. One of the risk factors for type 2 diabetes is insulin resistance. The hyperinsulinemic-euglycemic clamp (HEC) method is considered the gold standard test for the diagnosis of insulin resistance (IR), though it is expensive and less accessible in clinical practice [7]. Alternative markers, such as fasting plasma insulin (FPI) and the homeostasis model assessment of insulin resistance (HOMA-IR), are used to assess glycemic control and insulin sensitivity [8]. Recently, the triglyceride-glucose (TyG) index, calculated as Ln (fasting triglyceride (mg/dL) × fasting blood glucose (mg/dL) / 2), has emerged as a simple, cost-effective marker that predicts insulin resistance, metabolic syndrome, cardiovascular risk, and coronary artery calcification in addition to glycemic control [9]. Furthermore, the TyG index and its changes during a low-calorie diet were significant predictors of weight and fat loss, suggesting its potential as a biomarker for individualized response to dietary interventions in obesity [10]. A study conducted on three groups of normoglycemic, prediabetic, and diabetic patients in China revealed that the TyG index value had a negative correlation with pancreatic β-cell function with a cutoff value of 9.20 [11]. Moreover, the triglyceride glucose-body mass (TyG-BMI) index was significantly correlated with IR [12]. Recent study suggested that the TyG-BMI index is an effective predictor of all-cause and CVD mortality risks in diabetic patients [13].

On the other hand, the triglyceride to high-density lipoprotein cholesterol (TG/HDL-C) ratio serves as a marker of major adverse cardiovascular events, metabolic syndrome, and endothelial dysfunction, with a strong association with poor glycemic control, particularly in obese and insulin-resistant patients [9]. A longitudinal study showed that the elevated TG/HDL-C ratios increased the future risk of T2DM incidence among Chinese elderly [14]. Several studies have demonstrated significant associations between both the TyG index and TG/HDL ratios with poor glycemic control and cardiovascular risk, underscoring their potential as alternative biomarkers for glycemic control in T2DM patients [5,15]. This study aimed to investigate the predictive accuracy of the TyG index, TyG-BMI, and TG/HDL ratio for identifying poor glycemic control in Palestinian patients with type 2 diabetes.

## Materials and methods

Study design and study participants

A cross-sectional study was conducted between October 2023 and February 2024 at the Jericho Health Center, Palestine. The study was conducted according to the guidelines of the Declaration of Helsinki and approved by the Research Ethics Committee of Al-Quds University (333\REC\2023). Informed consent was obtained from all subjects involved in the study at the time of sampling. A total of 240 unrelated T2DM patients were recruited, aged ≥40 years, who had a previously established diagnosis of T2DM defined according to the WHO criteria (fasting plasma glucose (FPG) ≥126 mg/dl and/or treatment for diabetes). All individuals under 40 years, patients with T1DM, those with incomplete medical records, and pregnant women were excluded from the study. Demographic and clinical data, including age, gender, and diabetic complications, were obtained from medical records. BMI was calculated (weight in kilograms divided by the square of height in meters).

Biochemical measurements

Five milliliters of venous blood samples were taken from all study participants after an overnight fast. Two milliliters of the collected blood were placed into sterile ethylene diamine tetra acetic acid (EDTA) tubes to be used for the determination of HbA1c by the ARCHITECT C4000 instrument (Abbott Laboratories, Abbott Park, IL, US) and three milliliters were delivered in plain tubes and then centrifuged at 3000 rpm for 10 minutes, the obtained serum was used for the determination of: FPG, cholesterol, triglycerides, HDL-C, LDL-C by the ARCHITECT C4000 instrument at MOH Jericho Health Laboratory. Study participants who fulfilled the inclusion and exclusion criteria were divided into two groups: good glycemic control (HbA1c < 7%) and poor glycemic control (HbA1c ≥ 7%), based on American Diabetes Association (https://diabetes.org/about-diabetes/a1c) clinical guidelines that define this threshold as indicative of poor glycemic control in most nonpregnant adults with diabetes. The following formulas were used for the calculations:

TyG index = LN (triglyceride (mg/dL) × FPG (mg/dL) / 2)

TG/HDL = triglyceride(mg/dL) / HDL(mg/dL)

TyG-BMI = TyG index x BMI

Statistical analysis

The statistical package SPSS for Windows 23.0 (IBM Corp., Armonk, NY, US) was used for data analysis. A Shapiro-Wilk test was used to assess the normal distribution of the study variables. Non-normally distributed variables were expressed as median and interquartile range and compared using the Mann-Whitney U test and chi-squared test for categorical variables. Spearman's correlation analysis was used to assess the relationship of the TG/HDL ratio, TyG index, and TyG-BMI with clinical and biochemical parameters. The cutoff value for the TyG index and TyG-BMI was determined by analysis of the ROC curve. P <0.05 was considered statistically significant for comparisons.

## Results

Patient characteristics

A total of 240 diabetic patients were included in the study; 57% (n=137) were female and 43% (n=103) were male. The median (IQR) was 60 (53-66). Of them, 39% (n=94) had good glycemic control (HbA1C <7) and 61% (n=146) had poor glycemic control (HbA1C ≥7). Of all subjects, 15.8% had neuropathy, 7.9% had retinopathy, 2.9% had nephropathy, and 1.7% had diabetic foot.

The demographic and clinical data for all study participants are presented in Table 1.

**Table 1:** The demographic and clinical data for all study participants Continuous variables are presented as median (IQR). BMI, body mass index; SBP, systolic blood pressure; DBP, diastolic blood pressure; HbA1c, glycated hemoglobin; TC, total cholesterol; TG, triglyceride; LDL, low-density lipoprotein; HDL-C, high-density lipoprotein cholesterol; FPG, fasting plasma glucose; TyG index, triglyceride glucose index

Parameter	All subjects
Age (years)	60 (53-66)
Gender (male/female)	103/137
BMI (kg/m²)	30.1 (26.7-34.5)
SBP (mmHg)	135 (120-152)
DBP (mmHg)	74 (65-85)
HbA1C (%)	7.5 (6.4-8.7)
TC (mg/dL)	166 (145-192)
TG	120 (90-160)
LDL (mg/dL)	100 (79-112)
HDL-C (mg/dL)	40 (35-47)
FPG (mg/dL)	146 (124-194)
TyG Index	4.9 (4.7-5.1)
TG/BMI	150.1 (131-174.3
TG/HDL Ratio	3.1 (2.1-4.2)
Diabetic Complication	Percentage
Neuropathy	15.80%
Retinopathy	7.90%
Nephropathy	2.90%
Diabetic foot	1.70%

Comparison of clinical and biochemical parameters based on glycemic control

The demographic, clinical, and biochemical characteristics of the two groups were compared as shown in Table 2. The patients with poor glycemic control are younger than those with good glycemic control (median, IQR) (58.5 (50.75-65) vs. 64.0 (55.0-69.25; p=0.001).

The median (IQR) of fasting blood glucose was 182 (145-258), HbA1c 8.7 (7.9-10), TyG index 5.0 (4.8-5.3), and TyG-BMI 155 (133-178) were significantly higher in patients with poor glycemic control as compared to good glycemic control (p < 0.001) (Table 2). No statistically significant differences were observed in the median BMI, total cholesterol, LDL-C, triglycerides, systolic blood pressure, diastolic blood pressure, and TG/HDL ratio between the two groups (p >0.05). Furthermore, no significant differences were observed in the percentage of retinopathy, nephropathy, and diabetic foot complications between the two groups (p > 0.05). Notably, neuropathy was more prevalent among patients with poor glycemic control (19.9%) compared to those with good glycemic control (9.6%), p = 0.033 (Table 2).

**Table 2:** Comparison of demographic and biochemical variables in the studied groups Data are presented as median (IQR); p value was obtained by the Mann-Whitney U test (U) and chi-square (χ²) for categorical variables. SBP, systolic blood pressure; DBP, diastolic blood pressure; BMI, body mass index; FPG, fasting plasma glucose; HbA1c, glycated hemoglobin; TC, total cholesterol; TG, triglyceride; HDL-C, high-density lipoprotein cholesterol; LDL-C, low-density lipoprotein cholesterol; TyG index, triglyceride glucose index; TyG-BMI, product of TyG index and body mass index

Parameter	Good Glycemic Control (n=94)	Poor Glycemic Control (n=146)	U-Value	P-value
Age (years)	64 (55–69)	59 (51–65)	5136	<0.0001
BMI (kg/m²)	29.6 (26.7–34.4)	30.5 (26.9–35.0)	6694	0.749
SBP (mmHg)	132 (118–146)	137 (120–153)	6146	0.173
DBP (mmHg)	76 (66–87)	72 (64–85)	6460	0.444
HbA1C (%)	6.2 (6.0–6.6)	8.7 (7.9–10.0)	0	<0.0001
TC (mg/dL)	164 (136–191)	167 (147–192)	6525	0.522
TG	119 (91–152)	120 (90–177)	6579	0.59
LDL (mg/dL)	101 (74–117)	99 (82–117)	6606.5	0.626
HDL-C (mg/dL)	40 (35–45)	41 (35–48)	6438	0.419
FPG (mg/dL)	123 (105–136)	182 (145–258)	1691	<0.0001
TyG Index	4.79 (4.61–4.93)	5.03 (4.83–5.26)	3472.5	<0.0001
TG/BMI	141.8 (129.4–160.1)	155.2 (133.0–178.1)	5556	0.018
TG/HDL Ratio	3.2 (2.2–3.9)	3.1 (2.0–4.4)	6792	0.894
Parameter	n (%)	n (%)	χ²	P-value
Gender (female, n (%))	54 (57.4)	83 (56.8)	0.008	0.927
Neuropathy	9 (9.6)	29 (19.9)	4.54	0.033
Retinopathy	6 (6.4)	13 (8.9)	0.499	0.482
Nephropathy	2 (2.1)	5 (3.4)	0.34	0.708
Diabetic foot	2 (2.1)	2 (1.4)	0.2	0.654

Correlation of TyG, TyG-BMI indices, and TG/HDL ratios with study variables

Spearman’s rank correlation coefficient was used to assess the associations between study variables. A significant positive correlation was found between the TyG index and TyG-BMI index (r=0.412; p<0.001) and TG/HDL ratio (r=0.658; p<0.001). The TyG index and TyG-BMI both showed highly significant positive correlations with fasting blood sugar (FBS), HbA1c, and TG; and significant negative correlation with HDL (Table 3). The TyG-BMI index showed a strong positive correlation with BMI (r=0.939; p<0.001) and a weak positive correlation with systolic (r=0.204; p<0.002) and diastolic blood pressure (r=0.146; p<0.024). No correlation was found between the TG/HDL ratio and FBS and HbA1c (Table 3).

**Table 3:** Spearman's correlation of TyG, TyG-BMI indices, and TG/HDL with clinical and biochemical variables in T2DM patients **Correlation is highly significant at the 0.01 level. *Correlation is significant at the 0.05 level. P-values were calculated using a two-tailed test for Spearman’s rank correlation. SBP, systolic blood pressure; DBP, diastolic blood pressure; BMI, body mass index; FPG, fasting plasma glucose; HbA1c, glycated hemoglobin; TC, total cholesterol; TG, triglyceride; HDL-C, high-density lipoprotein cholesterol; LDL-C, low-density lipoprotein cholesterol; TyG index, triglyceride glucose index; TyG-BMI, product of TyG index and body mass index

	Variables	Correlation	p-Value
TyG Index	TyG_BMI	0.412**	<0.0001
	TG/HDL Ratio	0.658**	<0.0001
	Age	-0.078	0.23
	BMI	0.108	0.097
	Systolic BP	0.12	0.065
	Diastolic BP	0.021	0.743
	HbA1C	0.464**	<0.0001
	FPG	0.687**	<0.0001
	Cholesterol	0.254**	<0.0001
	TG	0.740**	<0.0001
	LDL	0.141*	0.029
	HDL	-0.200**	0.002
TyG_BMI	TG/HDL Ratio	0.340**	<0.0001
	Age	-0.029	0.657
	BMI	0.939**	<0.0001
	Systolic BP	0.204**	0.002
	Diastolic BP	0.146*	0.024
	HbA1C	0.185**	0.004
	FPG	0.231**	<0.0001
	Cholesterol	0.072	0.265
	TG	0.374**	<0.0001
	LDL	-0.006	0.928
	HDL	-0.149*	0.021
TG/HDL Ratio	Age	0.05	0.439
	BMI	0.118	0.067
	Systolic BP	0.085	0.192
	Diastolic BP	0.039	0.543
	HbA1C	-0.031	0.63
	FPG	0.076	0.242
	Cholesterol	0.08	0.218
	TG	0.906**	<0.0001
	LDL	0.043	0.503
	HDL	-0.630**	<0.0001

**Figure 1:**
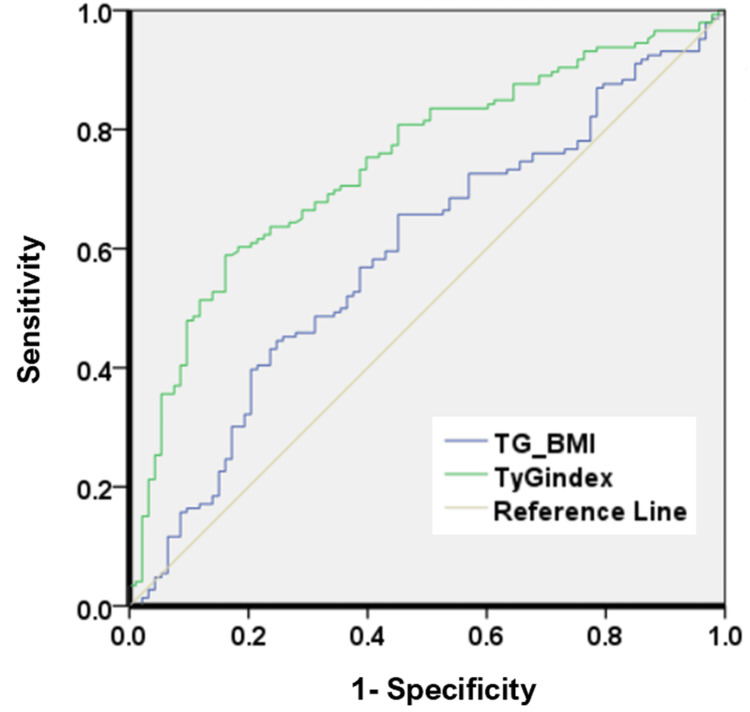
ROC curve analysis of the TyG and TyG-BMI indices to predict poor glycemic control in T2DM patients ROC, receiver operating characteristic; TyG, triglyceride-glucose; T2DM, type 2 diabetes mellitus

The ROC curve analysis was used to estimate the capability of the TyG index and TyG-BMI to predict poor glycemic control; the value of the AUC of the analysis is shown in Figure 1. The optimal cutoff value of the TyG index was 4.8 with a sensitivity of 81% and a specificity of 55% (AUC=0.744, 95% Cl: 0.681-0.807). While the optimal cutoff value for the TyG-BMI index was 162 with a sensitivity of 43.2% and a specificity of 76.3% (AUC=0.591, 95% Cl: 0.517-0.665) (Figure 1). A post hoc power analysis was conducted using the MedCalc online calculator (https://www.medcalc.org/calc/power-area-under-roc-curve.php) to confirm that the sample size of 240 (n=94 good glycemic vs. n=146 poor glycemic control) was adequate to achieve 99.9% power for the AUC of 0.744 at α = 0.05.

## Discussion

The rationale for using the TyG index is that IR is associated with impaired glucose uptake, increased triglyceride concentration, and reduced HDL-C concentration [16]. Several studies proposed that the TyG index could be a marker of IR with an excellent correlation with the gold standard euglycemic-hyperinsulinemic clamp test [17]. It has the advantage of being applicable in clinical practice since both triglyceride and glucose levels are routinely measured and the cost of these measurements is low [18]. TyG-derived indices, such as TyG-BMI, TyG-WC, and the TG/HDL ratio, also have been used to detect IR [19,20]. However, the real effectiveness of these markers remains questionable. Our findings indicate that the TyG index and TyG-BMI were significantly elevated in diabetic patients with poor glycemic control. Similarly, Selvi et al. illustrated that the TyG index and TyG-BMI were significantly correlated with HbA1c [15]. We observed that the TyG index showed significant positive correlations with HbA1c, FPG, and TG, supporting its potential role as a marker of metabolic dysfunction in diabetes. As the TyG index is calculated using both fasting TG and FPG, the elevated FPG directly contributes to an increased TyG value. A strong association between TyG and FPG reflects its biological relevance and suggests that the TyG index can be used as a surrogate marker for insulin resistance and metabolic dysfunction in diabetic patients. It is reported that hyperglycemia and insulin resistance trigger increased very low-density lipoprotein (VLDL) production and the release of chylomicron, which cause serum triglyceride levels to rise and to disrupt hepatic metabolism by stimulating glyconeogenesis and thus increase glucose production [21].

Also, we found significant negative correlations between the TyG index and TyG-BMI with HDL, indicating that as these indices increase, HDL levels tend to decrease. Low HDL is a known risk factor for cardiovascular disease, which is a common complication in poorly controlled diabetes. A retrospective research study conducted on Syrian refugees showed that the TyG index has the predictive ability to assess 10-year cardiovascular risk, determined by the Framingham risk score, with a sensitivity of 64.3% and a specificity of 75.0% [22].

In this study, we assessed the predictive performance of the TyG index and TyG-BMI for detecting poor glycemic control using ROC curve analysis; the AUC for the TyG index was significantly higher than that of the TyG-BMI, and the cut-off point of the TyG index was more sensitive than that of TyG-BMI. This is in accordance with Hameed et al.'s study, which revealed that the TyG index had a larger AUC in ROC analysis than TyG-BMI and was correlated with HbA1c [23]. Although BMI is generally associated with obesity and insulin resistance, it was not a useful predictor of poor glycemic control in our study cohort, this could be attributed to the homogeneity of our tested population in terms of BMI, as the median of all subjects was 30, which might have diminished the added value of BMI when combined with the TyG index as indicated by lower sensitivity and AUC observed for TyG-BMI compared to TyG index. A meta-analysis conducted on 25 studies assessed the performance of BMI to detect body adiposity, revealing that BMI cutoffs of > or =30 had a low sensitivity of 0.42 and a high specificity of 0.97 [24].

Several studies have investigated the diagnostic performance of the TyG index in identifying poor glycemic control. A Filipino study where a TyG index of >8.4 yielded high sensitivity (92.5%) but lower specificity (47.1%), highlighting its usefulness as a screening tool [25]. Similarly, a Bangladeshi study found a TyG Index of more than 4.762 had 84.5% sensitivity and 46.8% specificity for diagnosing IR [26] while a study on Mexican adults suggested that the TyG index is an alternative marker for assessing glycemic control using a cutoff of 4.93 with 80% sensitivity and 67% specificity [27]. However, a comprehensive systematic review evaluated the diagnostic accuracy of the TyG index against the hyperinsulinemic-euglycemic clamp. The study reported considerably different cutoffs, ranging from 4.55 to 4.78, with variable sensitivity and specificity, up to 96% and 99%, respectively [28]. These inconsistent results indicate the importance of selecting cutoff values tailored to clinical objectives, whether prioritizing early detection (sensitivity) or diagnostic precision (specificity).

Although the TG/HDL ratio has been used as a surrogate marker for insulin resistance, its sensitivity to changes in glycemic control may be limited as compared with indices that include glucose measurements. Our findings revealed no correlation between the TG/HDL ratio and FPG or HbA1C, and the median of the TG/HDL ratio was not statistically different in the two studied groups. In Iraqi populations, the TG/HDL ratio has shown a very good predictive value for insulin resistance among healthy adults [29], while in a study involving 125 African-American adults, no significant correlation between the TG/HDL ratio and insulin resistance was observed [30]. Moreover, a systematic review analyzed 32 cross-sectional studies to assess the performance of the TG/HDL ratio as an indicator of IR, revealing that the TG/HDL ratio had varied predictive power across ethnicities and genders [9].

This study has several limitations that should be acknowledged. First, the sample size was relatively small (240 participants), which may limit the generalizability of the findings. Additionally, it was conducted at a single center, reducing external validity. Also, the retrospective nature of our study further constrained the analysis, which relied on the information available in medical records. Thus, important clinical variables, such as waist circumference, duration of diabetes, levels of physical activity, and detailed medication history or comorbid conditions like hypertension, were not consistently recorded. Therefore, we could not account for the potential influence of these unmeasured confounders on glycemic control and metabolic markers. In line with previous studies and American Diabetes Association (ADA) clinical guidelines, we dichotomized HbA1c values at the 7.0% cutoff to assess the predictive performance of the TyG and TyG-BMI indices for glycemic control. Although this cutoff is clinically relevant, it may reduce sensitivity to trends across the HbA1c range. Future studies should be conducted using quartiles or continuous HbA1c values to investigate more detailed relationships. Despite these limitations, the statistical power for detecting the diagnostic performance of the TyG index based on AUC was high (>99.9%), indicating that our sample size was sufficient for the study analysis. To the best of our knowledge, this is the first study in Palestine to investigate the utility of the TyG and TyG-BMI indices in assessing glycemic control among patients with T2DM. Considering the rising prevalence of diabetes in the region and the limited availability of costly diagnostic tools, establishing a population-specific cutoff based on inexpensive and routinely available laboratory measures enhances its practical utility in everyday clinical settings where advanced testing is not available. This strategy could be adopted not only within the local healthcare setting but may also serve other lower- and middle-income countries facing similar resource limitations, thereby broadening the impact of our findings.

## Conclusions

In conclusion, our study demonstrates that the TyG index and TyG-BMI are significantly elevated in diabetic patients with poor glycemic control and show strong positive correlations with key glycemic and lipid parameters, including HbA1c, FPG, and triglycerides. A TyG index cutoff of 4.80, with higher sensitivity, may serve as a practical tool for identifying patients at risk of poor glycemic control, particularly in resource-limited settings. However, the low sensitivity of the TyG-BMI index and the lack of association with the TG/HDL ratio highlight the need for population-specific validation of surrogate markers for insulin resistance. Future prospective, multi-center studies with larger populations are needed to validate and standardize the cutoff values of the TyG index and TyG-BMI to be used in clinical practice in Palestine.
